# MR imaging features of benign retroperitoneal paragangliomas and schwannomas

**DOI:** 10.1186/s12883-017-0998-8

**Published:** 2018-01-04

**Authors:** Yanguang Shen, Yan Zhong, Haiyi Wang, Lu Ma, Yingwei Wang, Kun Zhang, Zhonghua Sun, Huiyi Ye

**Affiliations:** 1 Department of Radiology, Chinese PLA General Hospital, Fuxing Road 28, Box, Beijing, 100853 China; 2 Department of Radiology, Chinese Navy General Hospital, Beijing, China; 3 Department of Medical Radiation Sciences, Curtin University, Perth, 6102 Australia

**Keywords:** Paraganglioma, Schwannoma, Retroperitoneal space, Magnetic resonance imaging

## Abstract

**Background:**

To determine whether MRI feature analysis can differentiate benign retroperitoneal extra-adrenal paragangliomas and schwannomas.

**Methods:**

The MRI features of 50 patients with confirmed benign retroperitoneal extra-adrenal paragangliomas and schwannomas were retrospectively reviewed by two radiologists blinded to the histopathologic diagnosis. These features were compared between two types of tumours by use of the Mann-Whitney test and binary logistic regression. The patients’ clinical characteristics were reviewed.

**Results:**

Analysis of MRI images from 50 patients revealed no significant differences in the quantitative MRI features of lesion size, ratio of diameter and apparent diffusion coefficient. There were significant differences in the qualitative MRI features of location, necrosis, cysts and degree of tumour enhancement for two readers, with no significant differences in the other qualitative MRI features between these tumours. The combination of necrosis with degree of tumour enhancement during the arterial phase increased the probability that a retroperitoneal mass would represent retroperitoneal extra-adrenal paraganglioma as opposed to schwannoma.

**Conclusion:**

We have presented the largest series of MRI features of both benign retroperitoneal extra-adrenal paragangliomas and schwannomas. Some MRI features assist in the differentiation between these tumours, with imaging features consisting of necrosis and avid enhancement during the arterial phase, suggestive of retroperitoneal extra-adrenal paragangliomas.

## Background

Primary neurogenic tumours, which constitute 10% to 20% of primary retroperitoneal tumours, occur in a younger age group and are usually benign [1]. These tumours can be classified as ganglion cell origin, paraganglionic system origin (pheochromocytomas and paragangliomas), or nerve sheath origin (neurilemmomas, neurofibromas, neurofibromatosis, and malignant nerve sheath tumours). Schwannomas and extra-adrenal pheochromocytomas are the most common benign soft-tissue tumours occurring in the retroperitoneum [2, 3]; extra-adrenal pheochromocytomas account for 1–3%, and schwannomas account for 4% of retroperitoneal tumours [4, 5]. Retroperitoneal paragangliomas can be divided into functional and non-functional paragangliomas, and functional tumours are often associated with hypertension, tachycardia, headache and diaphoresis [6]. However, non-functional paragangliomas can be completely clinically silent (they are referred to as “incidentalomas”). Since retroperitoneal benign schwannomas are usually asymptomatic, diagnostic difficulties are often encountered for differentiating retroperitoneal paragangliomas from schwannomas due to their nonspecific clinical and imaging features. However, any physical contact with these silent paragangliomas can precipitate cardiac arrhythmias and malignant hypertension [7]. Now, more than ever, urologists and radiologists should understand the imaging appearances of paragangliomas, as differential diagnosis between them is clinically essential in decision making on a therapeutic strategy.

To the best of our knowledge, description of the MRI features of retroperitoneal extra-adrenal paragangliomas and schwannomas has been reported on small sample sizes in the literature, and no joint assessment has been performed for the imaging appearances of these two similar entities, which may be expected to have overlapping imaging findings in view of their common pathologic characteristics. Therefore, the purpose of our study was to retrospectively analyse the MRI imaging differences of benign retroperitoneal extra-adrenal paragangliomas and schwannomas, particularly for those with non-functional paragangliomas.

## Methods

### Patients

This retrospective study was approved by our institutional review board with waiver of informed consent due to the retrospective nature. Between July 2008 and February 2016, retroperitoneal extra-adrenal paragangliomas and schwannomas were identified within the radiology databases and were confirmed by surgical resection and pathological findings in 50 patients who had undergone preoperative MRI. Of these 50 patients, 20 had undergone preoperative CT (13 paraganglioma and 7 schwannoma patients). All patients’ medical histories were reviewed. In all, 50 patients (30 men and 20 women, mean age: 44.3 ± 12.1 years, age range: 17–79 years old) had a total of 50 tumours: 24 benign retroperitoneal extra-adrenal paragangliomas and 26 benign retroperitoneal schwannomas.

### Magnetic resonance imaging protocol

MRI examinations were performed with a 1.5-T system (*n* = 5, Signa HDXT, GE Healthcare), 3.0-T system (*n* = 28, Signa EXCITE; GE Healthcare, Milwaukee, WI, USA) and a 3.0-T system (*n* = 17, Discovery 750, GE Healthcare, Milwaukee, WI, USA). A surface phased-array coil was used, with all patients in the supine position. Respiratory-triggered transverse and coronal T2-weighted fast spin-echo sequences were initially performed, followed by transverse T1-weighted dual-echo in-phase and out-of-phase sequences and three-dimensional fat-saturated T1-weighted dynamic contrast-enhanced sequences performed during suspended respiration. Transverse breath-hold diffusion-weighted imaging (DWI) was obtained using a single-shot, spin-echo echo-planar sequence prior to the administration of contrast material with tri-directional gradients and two sets of b values: 0 and 800 s/mm^2^. A 15-mL bolus of contrast medium (Gadobenate dimeglumine, MultiHance; Bracco Sine, Shanghai, China) was injected intravenously at a flow rate of 2 mL/s using a power injector (Spectris; MedRad, Warrendale, PA, USA), followed by a 20-mL saline flush. Dynamic contrast-enhanced MRI (DCE-MRI) was performed in the transverse plane at baseline (precontrast) and during the arterial, venous and delayed phases.

The MR imaging parameters for T2-w FSE images were as follows: infinite/90–105 ms (repetition time ms/echo time ms); field of view (FOV), 36–44 cm; section thickness, 5 mm; intersection gap, 1 mm; and matrix, 320 × 224. The imaging parameters for T1-w dual-echo images were as follows: 260/(2.2–2.5; 5.5–5.8) ms; FOV, 36–44 cm; section thickness, 5 mm; intersection gap, 1 mm; and matrix, 256 × 192. The scanning protocol of DWI was as follows: 5400/50–60 ms; FOV, 36–40 cm; flip angle, 90°; matrix, 128 × 128; section thickness, 5 mm; intersection gap, 1 mm; all directions; one signal acquired, b values of 0, 800 s/mm^2^. The parameters for three-dimensional (3D) DCE-MRI sequences were as follows: 3.0–3.9/1.2–1.6 ms; FOV, 34–40 cm; section thickness, 5 mm; interpolated section thickness, 2.5 mm; and matrix, 288 × 224.

#### Imaging features analysis

Two radiologists who had 10 and 5 years of experience in the interpretation of abdominal MR images independently reviewed all images. Readers who were blinded to the histologic diagnoses of the lesions evaluated and recorded each lesion for the presence of each of the following features [8, 9].

##### Quantitative MR image feature analysis

(A) Tumour maximum size: the maximum size of each lesion was measured at its single largest diameter in three planes. (B) Ratio of diameter: two readers independently measured the maximum transverse diameter (TD) and longitudinal diameter (LD) of the mass in the coronal section. The means of maximum TD and LD of the tumour in the coronal section were recorded. Each measurement was conducted three times, with the mean value used as the final value to avoid intra- and inter-observer disagreements. Ratios of diameter were determined by the following equations: Ratio of diameter = Mean TD/ Mean LD. (C) Apparent diffusion coefficient (ADC) values: ADC maps were auto-generated. The region of interest (ROI) was placed corresponding to the most obvious enhancing region of the retroperitoneal tumours in the arterial phase according to visual assessment, with the aim of avoiding necrosis, haemorrhage and cystic changes (which are defined as those parts of the lesions showing no enhancement on DCE-MR images) and was performed using an Advantage Workstation (Advantage Workstation, version 4.6, GE Healthcare, Bue, France). In each patient and for each tumour, three ROIs, each measuring 30–60 mm^2^, were drawn on three target anatomical structures. The mean ADC values in ROIs on three targets were calculated for each patient.

##### Qualitative MR image feature analysis

(1) Position and peripheral location: The lesion was assessed according to whether it was located in the right paravertebral region or the left paravertebral region (near the spinal column and psoas muscle, adrenal region or kidney); anterior to the vertebrae (close to the abdominal aorta and the inferior vena cava, the origin of the inferior mesenteric artery or peripancreatic location); or in the pelvic cavity. (2) Shape: Tumours were round or oval/irregular. (3) Margin: Tumours were well-defined or partly poorly defined. (4) Microscopic lipid: There was an area of signal loss in the lesion on out-of-phase T1-weighted images. (5) Subacute haemorrhage: There were areas of increased T1 signal intensity on unenhanced fat suppressed T1-weighted images. (6) Cystic degeneration: Represented by areas with signal intensities (SIs) equal to that of cerebrospinal fluid on T2-weighted images; low SI on T1-weighted images; lack of enhancement; and lobulated morphology. (7) Necrosis: Represented by high SI on T2-weighted images, although not as high as the signal of cerebrospinal fluid; low SI on T1-weighted images; lack of enhancement; and central location within the tumour. (8) Degree of tumour enhancement: Subjective assessments of the MR imaging degree of mass enhancement compared with that of the renal cortex (avid enhancement, moderate enhancement or slight enhancement) were performed on Gd-enhanced MR images acquired during the arterial phase. (9) Other MR imaging features: Calcification, smooth expansion of a sacral nerve root exit foramen, fluid-fluid level, and subjective assessment of the MR imaging degree and pattern of mass enhancement during the venous and delayed phases.

### Pathologic diagnosis

All specimens were retrospectively examined by two uropathologists who were unaware of the MRI findings (10 years of experience in uropathology) in consensus.

#### Statistical analysis

Continuous variables are expressed as the means ± SD and were analysed by independent t-tests for normally distributed data or Mann-Whitney tests for non-normally distributed data. For the qualitative variables, the Chi square test was used to compare the sample proportions of the two groups. Generalized estimating equations based on a binary logistic regression model were used to determine whether lesion type (retroperitoneal extra-adrenal paragangliomas or schwannomas) was associated with any of the individual binary factors. In this context, stepwise variable selection was performed to determine whether the combination of two or more of the aforementioned imaging features represented a significant independent predictor of schwannoma or paraganglioma. These tests were performed separately for each reader. Kappa coefficients were not used for this determination because the very high prevalence rates of certain imaging features for many of the binary factors was expected to produce misleadingly low values [8, 10]. All reported *p* values are two-sided and considered statistically significant when less than 0.05. SPSS version 19.0 software (IBM Corporation, Armonk, NY, USA) was used for all computations.

## Results

### Demographic data and clinical characteristics

**Fig. 1:**
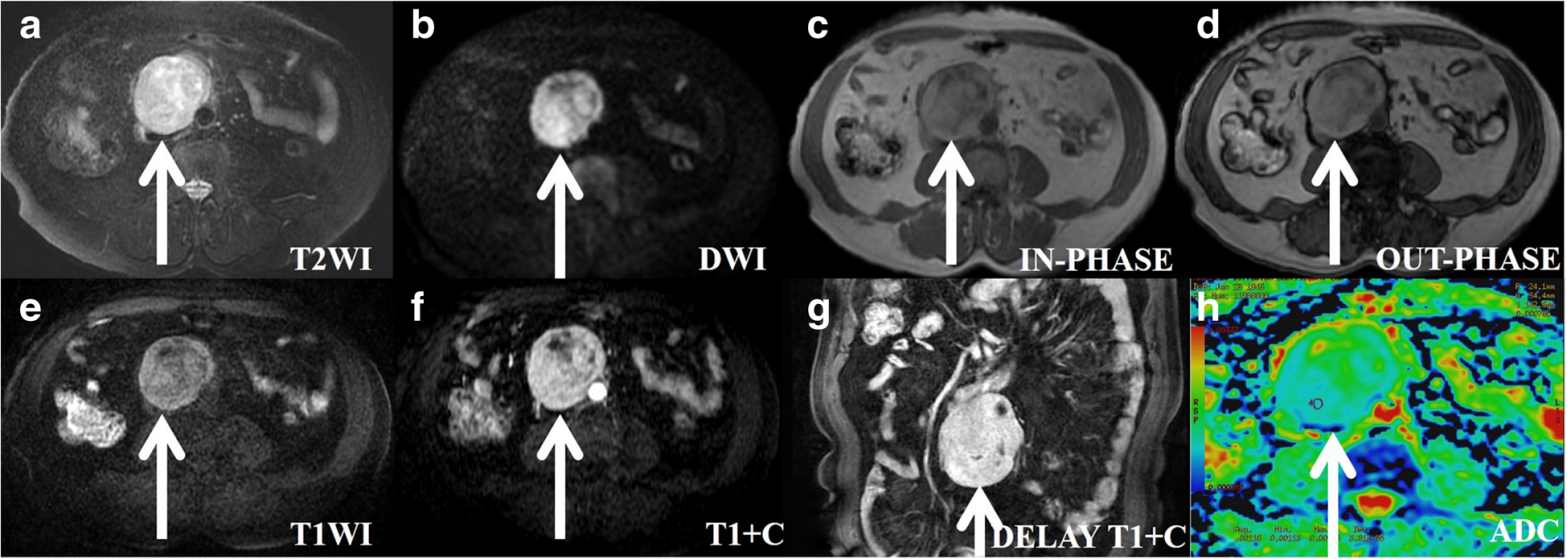
A patient (age: 60–69) with hypertension and a histologically proven benign retroperitoneal paraganglioma in the right prevertebral region (between the inferior vena cava and aorta). The transverse and longitudinal diameters of the tumour are 8 cm and 9.25 cm, respectively. **a** On axial T2WI, the tumour appears with high signal intensity compared with the gluteal muscles. The intratumoural cystic areas are higher in signal intensity. **b** On axial DWI, the tumour appears circular and nodular, with high signal intensity. **c**, (**d**) and (**e**) In T1WI (in-phase, out-of-phase and pre-scanned imaging), part of the tumour signal is slightly lower than that of the gluteal muscle, and most of it is slightly higher than that of the gluteal muscle. **f** On contrast T1WI during the aerial phase, the tumour exhibits obvious and inhomogeneous enhancement. **g** On coronal T1WI during the delay phase, the tumour exhibits obvious and inhomogeneousenhancement. **h** On ADC imaging, the mean ADC value of the ROI of the tumour is 0.00119 mm^2^/s

**Fig. 2:**
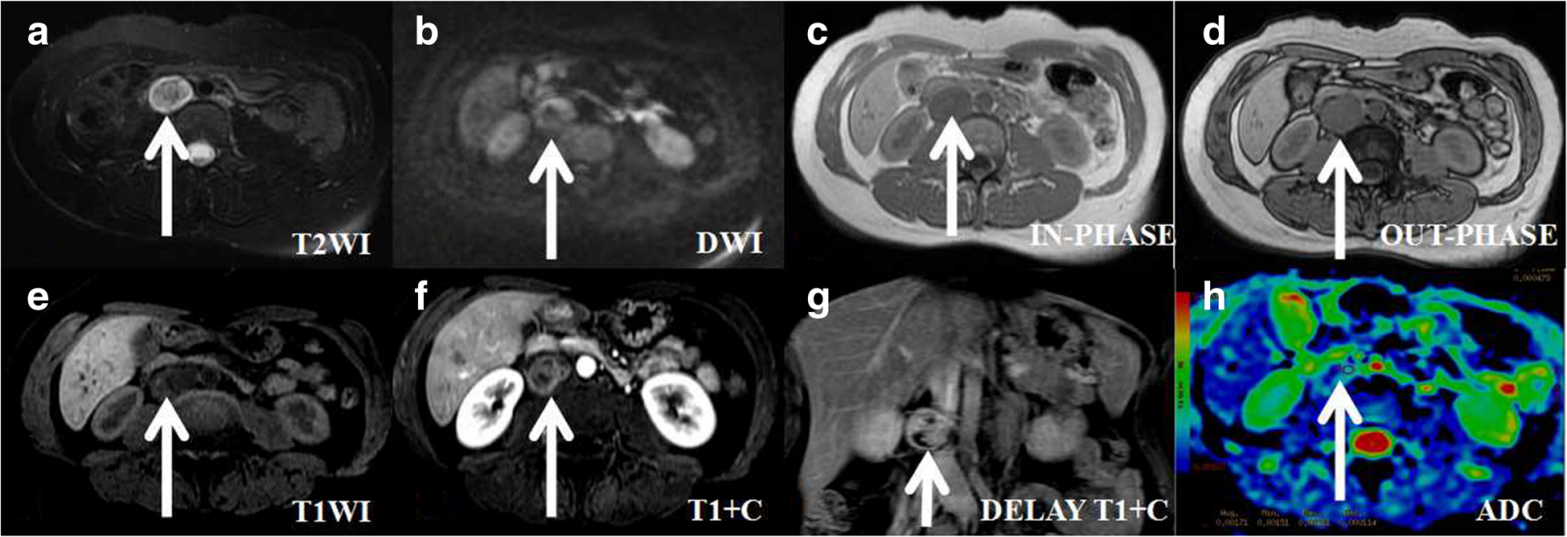
An asymptomaticpatient (age: 30–39) with a histologically proven benign retroperitoneal schwannoma. The transverse and longitudinal diameters of the tumour are 2.19 cm and 3.51 cm, respectively. **a** On axial T2-weighted imaging, the tumour presents with heterogeneous signal intensity compared with the gluteal muscles. **b** On axial DWI, the tumour appears circular, with high signal intensity. **c**, (**d**) On T1-weighted imaging (in-phase, out-of-phase imaging), the tumour’s signal is slightly lower and isointense compared with that of the gluteal muscle. The intratumoural microscopic fat areas are slightly lower in signal intensity on out-of-phase imaging. **e** On T1-weighted imaging (pre-scanned imaging), the tumour’s signal is slightly lower, with isointense and hyperintense spots compared with that of gluteal muscle. The intratumoural subacute haemorrhage areas present as hyperintense spots in signal intensity on pre-scanned imaging. **f** On contrast T1-weighted imaging during the aerial phase, the tumour presents as slightly enhanced and inhomogeneous. **g** On coronal T1-weighted imaging during the delay phase, the tumour presents as moderately enhanced and inhomogeneous. **h** On ADC imaging, the mean ADC value of the ROI of the tumour is 0.00171 mm^2^/s

In this study, a total of 50 lesions in 50 patients were identified for inclusion in the analysis, consisting of 24 retroperitoneal extra-adrenal paragangliomas (Fig. 1) and 26 retroperitoneal schwannomas (Fig. 2). All presenting clinical characteristics of these patients are summarized in Table 1. A total of 30 tumours were incidentally found in 50 patients. The 24-h urinary vanilmandelic acid and urinary catecholamine concentrations were measured for 22 of the patients, of which 9 were positive. No patient had a medical history of neurofibromatosis. The tumours were fully excised in all cases, with clear resection margins. The final histologic diagnosis was obtained by laparoscopic surgery (15 lesions), robot-assisted laparoscopy (11 lesions), and laparotomy (24 lesions).

**Table 1:** Patient demographics and clinical characteristics

Characteristic	Paragangliomas group (*n* = 24)	Schwannoma group (*n* = 26)	*P*-value
No. of patients (female)	24 (12)	26 (8)	–
^a^Age (years)	48.04 ± 9.70	40.88 ± 13.29	0.036
Hypertension (n)	11	1	0.001
CA-related symptoms (n)	3	0	0.001
Symptomless(n)	10	20	0.001
VMA/24 h (Positive)	22 (9)	0	0.001
CA/24 h (Positive)	22 (9)	0	0.001
^131^I–MIBG positive	9	0	0.001
other symptoms			
Abdominal mass (n)	2	4	–
Lumbago (n)	3	2	–
dysuria (n)	0	1	–
Resection of tumor			
Laparoscopic surgery (n)	9	6	–
Robot-assisted laparoscopic (n)	10	1	–
Laparotomy (n)	5	19	–

^a^Values are mean values ± standard deviations. *P* values were calculated by using t test

Other data are numbers of patients (n). *P* values were calculated by using x^2^ test. *CA-related symptoms* catecholamine-related symptoms, *VMA* Vanilmandelic acid, *CA* Catecholamine, *MIBG* Metaiodobenzylguanidine scintigraphy

### Findings of the quantitative MR imaging features

The presenting quantitative MR imaging characteristics of mean maximum lesion size, ratio of diameter and ADC values of the 24 retroperitoneal extra-adrenal paragangliomas and 26 schwannomas are summarized in Table 2, with no significant differences in the assessments by two reviewers. Of the 50 lesions, the maximum diameter was greater than 5 cm in 34 cases (17 retroperitoneal extra-adrenal paragangliomas and 17 schwannomas), while in the remaining 16 cases, the maximum diameter was less than 5 cm (7 paragangliomas and 9 schwannomas).

**Table 2:** Quantitative characteristics of retroperitoneal extra-adrenal paragangliomas and schwannoma

Item	Reader 1			Reader 2		
	Paragangliomas (*n* = 24)	Schwannoma (*n* = 26)	*P*-value	Paragangliomas (*n* = 24)	Schwannoma (*n* = 26)	*P*-value
Tumor maximum size (cm)	5.61 ± 2.11	6.35 ± 3.24	0.350	5.70 ± 2.10	6.19 ± 3.04	0.521
Ratio of diameter (TD/LD)	0.878 ± 0.145	0.831 ± 0.148	0.175	0.855 ± 0.144	0.802 ± 0.151	0.212
Mean ADC value (×10^−3^ mm^2^/s)	1.541 ± 0.425	1.614 ± 0.345	0.508	1.565 ± 0.425	1.651 ± 0.322	0.421

Values are Mean values ± standard deviations. *P* values were calculated by using t test

Ratios of diameter = Mean TD(transverse diameter)/ Mean LD(longitudinal diameter). *ADC* Apparent Diffusion Coefficient

### Findings of the qualitative MR imaging features

(1) There were statistically significant differences in terms of lesion location, necrosis, cystic degeneration and degree of tumour enhancement for both readers (*p* = 0.000–0.011, 0.000–0.019 for readers 1 and 2, respectively), while there were no statistically significant differences between retroperitoneal extra-adrenal paragangliomas and schwannomas in terms of shape, boundaries, microscopic fat, and subacute haemorrhage findings for both readers (*p* = 0.164–0.589, 0.271–1.0 for readers 1 and 2, respectively) (Table 3).

**Table 3:** Frequency of assessed imaging features in retroperitoneal extra-adrenal paragangliomas and schwannoma

Imaging Feature	Reader 1			Reader 2		
	Paragangliomas (*n* = 24)	Schwannoma (*n* = 26)	*P*-value	Paragangliomas (*n* = 24)	Schwannoma (*n* = 26)	*P*-value
Location						
Right paravertebral region	8.33(2/24)	50(13/26)	0.001	8.33(2/24)	50(13/26)	0.001
Left paravertebral region	37.5(9/24)	11.53(3/26)		41.67(10/24)	11.54(3/26)	
Prevertebral region	54.17(13/24)	23.08(6/26)		50(12/24)	23.08(6/26)	
Pelvic cavity	0(0)	15.38(4/26)		0(0)	15.38(4/26)	
Shape						
Round or oval	83.33(20/24)	92.31(24/26)	0.589	87.5(21/24)	84.62(22/26)	1.000
Irregular	16.67(4/24)	7.69(2/26)		12.5(3/24)	15.38(4/26)	
Boundaries						
Well-defined	66.67(16/24)	88.46(23/26)	0.063	70.83(17/24)	84.62(22/26)	0.240
Partly poorly defined	33.33(8/24)	11.54(3/26)		29.17(7/24)	15.38(4/26)	
Microscopic fat						
YES	12.5(3/24)	23.08(6/26)	0.546	16.67(4/24)	15.38(4/26)	1.000
NO	87.5(21/24)	76.92(20/26)		83.33(20/24)	84.62(22/26)	
Subacute hemorrhage						
YES	54.17(13/24)	34.62(9/26)	0.164	50(12/24)	34.62(9/26)	0.271
NO	45.83(11/24)	65.38(17/26)		50(12/24)	65.38(17/26)	
Necrosis						
YES	75(18/24)	34.62(9/26)	0.004	70.83(17/24)	34.62(9/26)	0.010
NO	25(6/24)	65.38(17/26)		29.17(7/24)	65.38(17/26)	
Cysts						
YES	62.5(15/24)	92.31(24/26)	0.011	66.67(16/24)	96.15(25/26)	0.019
NO	37.5(9/24)	7.69(2/26)		33.33(8/24)	3.85(1/26)	
Degree of tumor enhancemen						
Avid enhancement	41.67(10/24)	3.85(1/26)	<0.001	33.33(8/24)	7.69(2/26)	<0.001
Moderate enhancement	41.67(10/24)	19.23(5/26)		50(12/24)	15.38(4/26)	
Slight enhancement	16.67(4/24)	76.92(20/26)		16.67(4/24)	76.92(20/26)	

Data are percentages with raw numbers of patients (n) in parenthesis

*P* values were calculated by using x^2^ test

(2) Concordance of the two readers for each of the assessed binary features was good to excellent, ranging from 52% to 98% for all features (Table 4).

**Table 4:** Comparison of concordance between readers for each assessed qualitative imaging feature

Attribute	Concordance
Location	98(49/50)
Shape	94(47/50)
Tumor boundaries	96(48/50)
Microscopic fat	94(47/50)
Subacute hemorrhage	92(46/50)
Necrosis	52(26/50)
Cystic degeneration	76(38/50)
Degree of tumor enhancement	94(47/50)

Values are percentages with raw numbers of patients (n) in parenthesis

(3) Only two features remained statistically significant in the stepwise multivariate logistic regression model: necrosis and degree of tumour enhancement. The combination of necrosis with degree of tumour enhancement during the arterial phase increased the probability that a retroperitoneal mass would represent extra-adrenal paraganglioma versus schwannoma, with diagnostic accuracies (c statistic or area under the curve-AUC) of 0.893 (reader 1) and 0.853 (reader 2)and with 95% confidence intervals (CIs) of 0.807–0.978 for reader 1 and 0.748–0.9579 for reader 2 (Table 5).

**Table 5:** Stepwise multivariate logistic regression analysis relating statistically significant MR imaging features to retroperitoneal extra-adrenal paragangliomas

Readers and Features	Adjusted Odds Ratio
Radiologist 1	
Necrosis	0.101 (0.017, 0.595)
Degree of tumor enhancement	0.097 (0.026, 0.354)
Radiologist 2	
Necrosis	0.148 (0.032, 0.684)
Degree of tumor enhancement	0.150 (0.048, 0.472)

The model c statistic (Area Under Curve, AUC) was 0.893 (95%CI was 0.807–0.978) for reader 1, AUC = 0.853 (95%CI was 0.748–0.957) for reader 2. Numbers in parentheses are 95% confidence intervals

(4) No retroperitoneal extra-adrenal paragangliomas were identified in the pelvis, though 4 schwannomas were identified in the pelvis, with 1 case located in the medial iliac arterial bifurcation, 2 cases in the wall of the basin, and another in the anterior sacral space. Approximately 54.17% of the paragangliomas were located in the prevertebral region, which is close to the aorta and the inferior vena cava. However, 50% of schwannomas were in the right paravertebral region (2 cases in the renal portal area, 10 cases in lumbar major muscles, and 1 case in the adrenal region).

### Findings of other MR imaging features

First, only small sub-centimetre flecks of calcification were seen in 2 schwannomas and 1 paraganglioma. Second, only two schwannomas were shown to demonstrate smooth expansion of a sacral nerve root exit foramen without including bony destruction of the sacrum. Third, all tumours showed inhomogeneous enhancement following gadolinium administration, with a non-enhancing component showing a fluid signal or necrotic component and peripheral enhancement of the solid elements. Only one paraganglioma had a fluid-fluid level inside the lesion. The degrees of enhancement of 21 paragangliomas and 26 schwannomas showed continuous signal increases in mass in the venous and delayed phases (the persistent pattern), and only 3 paragangliomas showed “washout” of signal intensity.

## Discussion

Retroperitoneal extra-adrenal paragangliomas and schwannomas confined to the retroperitoneum are frequently encountered in clinical practice, and diagnostic difficulties are often encountered [11]. To the best of our knowledge, this study represents the largest series to date to describe the MRI features of both tumours, with the aim of differentiating them due to overlapping clinical, MR imaging and histologic features.

Paragangliomas are usually described on MRI as masses having characteristic high-signal intensity or a light bulb bright signal on T2WI with the use of fat suppression [12], which is used to differentiate them from other tumours, but further studies have proposed that this feature is neither specific nor sensitive and have indicated that the use of this sign leads to the misdiagnosis of paragangliomas in up to 35% of cases [13, 14]. The MRI characteristics of benign retroperitoneal schwannomas include hypointensity on T1WI and hyperintensity on T2WI [15, 16], and neither is specific. However, in our results, the MRI features consisting of tumour location, necrosis and tumour enhancement showed significant differences between these two retroperitoneal tumours.

More than 50% of paragangliomas were situated in the prevertebral region close to the inferior vena cava and aorta following the aorto-sympathetic chain, results that were consistent with other reports [17]. However, schwannomas were usually located in the paravertebral region and, less commonly, adjacent to the kidney, pre-sacral space, and abdominal wall [6]. Although the appearances overlapped with the reported appearances of many retroperitoneal tumours [18],no statistically significant differences were found in the stepwise multivariate logistic regression model in our study. One obvious specific feature of 3 schwannomas was smooth expansion of a nerve root exit foramen without showing bony destruction, which is highly suggestive of retroperitoneal schwannomas [19].

DCE-MRI has also been employed for tumour detection and characterization. In this study, 80% of retroperitoneal extra-adrenal paragangliomas exhibited strong initial signal increases during the arterial phase; however, 76.92% of schwannomas mostly demonstrated slow initial signal enhancement. These results are consistent with other studies [20, 21]. It should be noted that a pattern of continuous signal increase of masses was common in these tumours in the venous and delayed phases and was not helpful to distinguish paragangliomas from schwannomas. Necrotic change was noted in more than 70% of retroperitoneal extra-adrenal paragangliomas but in only 34.62% of schwannomas in our study. Necrotic changes tend to occur as paragangliomas increase in size [18]. Further research showed that a combination of avid enhancement with necrosis provided diagnostic accuracies of 0.853 and 0.893 for the diagnosis of retroperitoneal extra-adrenal paragangliomas in our series. In other words, these findings allowed the differentiation of paragangliomas from schwannomas: avid enhancement and necrosis were predictive of paragangliomas, while slight enhancement was correlated with schwannomas. Paragangliomas are characteristically highly vascular neoplasms and an abundant capillarynetwork,and may have precarious microcirculation because of high levels of tissue vasoconstrictor substances. These histologic features can cause spontaneous massive intratumoural haemorrhage and necrotic degeneration, resulting in the formation of a pseudocyst, and exhibits marked and early enhancement in DCE-MRI [22, 23]. Further, Sahdev et al. reported that necrotic changes were observed in more than 70% of retroperitoneal extra-adrenal paragangliomas and tended to occur as paragangliomas increased in size [18]. However, many theories attempt to explain the degeneration cystic schwannoma. One theory involves the degeneration of Antoni B areas leading to cyst formation, while progressing in size; another theory holds that with increasing tumor central ischemic necrosis occurs that causes cysts within the tumor [24, 25]. On histopathological examination, Antoni B area is a myxoid component.

No significant differences were shown between the mean lesion sizes and mean ADC values of these two types of tumours in our study. This study suggested that the ADC quantitative assessment could not provide significant value for the differential diagnosis of both of the tumours. We found that the mean ADC values of these tumours were greater than the mean ADC values of neck paragangliomas and schwannomas [26, 27].

Evidence of degeneration, which includes cysts, subacute haemorrhage and microscopic fat, was common for retroperitoneal extra-adrenal paragangliomas and schwannomas, and all tended to be observed [28]. Takatera et al. [19] reported that 66% retroperitoneal schwannomas showed cystic degeneration, while schwannomas demonstrated an exceptional risk of degeneration [11, 29]. In this study, more than 90% of retroperitoneal schwannomas showed the same feature, which was only found in 62.5% of retroperitoneal extra-adrenal paragangliomas, highlighting that fact that this feature is helpful in the differential diagnosis. Literature studies have reported that haemorrhagic portions can be seen in paragangliomas and schwannomas [30, 16]; however, they did not report that the feature was able to distinguish these tumours. The feature of subacute haemorrhage was found in 50% of retroperitoneal extra-adrenal paragangliomas and 34.62% of schwannomas, and only one case showed a fluid-fluid sign in paragangliomas. In addition, microscopic fat was not reported in the literature, and there was no obvious specificity. Calcifications can occur in all types of neurogenic tumours. In our study, calcifications were seen in only 3 lesions, without showing any obvious specificity. However, some authors have reported that punctate calcifications can be seen in retroperitoneum extra-adrenal paragangliomas, along with punctuate or curvilinear calcifications along the walls of masses in schwannomas [31].

There are several limitations in our study. **First**, this was a retrospective study with a relatively small sample size for benign retroperitoneal extra-adrenal paragangliomas and schwannomas, reflecting the low incidence rates of these tumours. The imaging features of our patients were similar to those described in other radiological series, and the small number of cases reflected the rarity of the tumours. **Second**, due to the study’s retrospective nature, two different field strengths of magnetic resonance scanners were used. Although we demonstrated that field strength had no effect on ADC measurements of renal tumours between 1.5 T and 3.0 T, we did not include many retroperitoneal tumours. In addition, ADCs of various kinds of retroperitoneal lesions should be compared between 1.5 T and 3.0 T. **Third**, lesions presented with predominantly cystic changes, haemorrhage and necrosis, which may affect ADC values or signal intensity measurements. **Finally**, there was only one malignant schwannoma and no malignant paragangliomas. We excluded these tumours from the analysis and did not perform any further studies of benign and malignant retroperitoneal tumours.

## Conclusions

In summary, in this study, we present the largest series of radiological studies of benign retroperitoneal extra-adrenal paragangliomas and schwannomas. These tumours are often found incidentally or present with vague and nonspecific symptoms. They are rare retroperitoneal neoplasms, usually presenting as large ovoid or spherical masses with smooth, well-defined borders and do not invade or obstruct adjacent structures. The combination of avid enhancement with necrosis, clinical CA-related symptoms, positive VMA/24 h, positive CA/24 h and positive 131I–MIBG provided diagnostic accuracy for the diagnosis of retroperitoneal extra-adrenal paragangliomas in our series. When these features are correctly recognized, there should be a high level of suspicion for paragangliomas.

## Abbreviations

ADC: Apparent diffusion coefficient
AUC: Area under the curve
CA: Catecholamine
DCE-MRI: Dynamic contrast-enhanced MRI
FOV: Field of view
LD: Longitudinal diameter
MIBG: Metaiodobenzylguanidine scintigraphy
MRI: Magnetic resonance imaging
ROI: Region of interest
T1WI: T1-weighted imaging
T2WI: T2-weighted imaging
TD: Transverse diameter
VMA: Vanilmandelic acid
